# Diagnostic accuracy of ^18^F-FDG PET/CT in muscle-invasive bladder cancer: rationale and design of the MIBC-PET study

**DOI:** 10.3389/fmed.2026.1792243

**Published:** 2026-03-12

**Authors:** Luca Urso, Francesco Feletti, Federica Lancia, Francesca Canella, Corrado Cittanti, Ilham Badrane, Sara Adamantiadis, Licia Uccelli, Antonella Iudicello, Alberto Nieri, Matteo Caracciolo, Ilaria Rambaldi, Riccardo Bisi, Luigi Manco, Ilaria Bagni, Massimo Guidoboni, Luana Calabrò, Carmelo Ippolito, Melchiore Giganti, Mirco Bartolomei

**Affiliations:** 1 Department of Translational Medicine, University of Ferrara, Ferrara, Italy; 2 Nuclear Medicine Unit, Onco-Hematology Department, University Hospital of Ferrara, Ferrara, Italy; 3 Unità Operativa Radiologia, Ospedale S. Maria delle Croci Ravenna, Azienda USL della Romagna, Ravenna, Italy; 4 Oncology Unit, Onco-Hematology Department, University Hospital of Ferrara, Ferrara, Italy; 5 Unità Operativa Radiologia Universitaria, Azienda Ospedaliero-Universitaria “S. Anna”, Ferrara, Italy; 6 Medical Physics Unit, University Hospital of Ferrara, Ferrara, Italy; 7 Anatomic Pathology Unit, University Hospital of Ferrara, Ferrara, Italy; 8 Urology Unit, Surgical Department, University Hospital of Ferrara, Ferrara, Italy

**Keywords:** ^18^F-FDG, artificial intelligence, diagnostic accuracy, FDG PET, muscle-invasive bladder cancer, neoadjuvant chemotherapy, PET/CT, radiomics

## Abstract

**Introduction:**

Muscle-invasive bladder cancer (MIBC) is associated with poor prognosis. Current staging relies on CT and MRI, but both show limited sensitivity for nodal and distant metastases. [^18^F]FDG PET/CT has emerged as a promising tool for staging and response assessment, yet its role remains undefined.

**Methods and analysis:**

MIBC-PET is a prospective, monocentric, no-profit study with paired imaging comparison enrolling 60 patients with newly diagnosed, histologically confirmed high-grade MIBC. All patients undergo baseline staging with [^18^F]FDG PET/CT and contrast-enhanced CT (ceCT) of chest, abdomen, and pelvis. A second [^18^F]FDG PET/CT and CT are performed after two cycles of neoadjuvant therapy. [^18^F]FDG PET/CT includes both standard delayed imaging and an early dynamic pelvic acquisition to overcome urinary tracer interference. The primary endpoints are (1) diagnostic accuracy of [^18^F]FDG PET/CT vs. ceCT for baseline staging, and (2) accuracy of [^18^F]FDG PET/CT in predicting pathological response to neoadjuvant chemotherapy. Secondary endpoints include validation of early dynamic [^18^F]FDG PET/CT for local staging and radiomic/AI-based predictive modeling.

**Ethics and dissemination:**

The study complies with the Declaration of Helsinki and Good Clinical Practice guidelines. The study was approved by the local ethical committee (CE-AVEC 109-2024-Oss-AOUFe – P.I. Dr. Luca Urso, MD). Results will inform the integration of [^18^F]FDG PET/CT into diagnostic algorithms for MIBC.

**Trial registration:**

ClinicalTrials.gov, identifier: not yet assigned. Local trial registration code: CE-AVEC 109-2024-Oss-AOUFe.

## 1 Introduction

Bladder cancer ranks as the 10th most common malignancy worldwide, with approximately 30% of patients presenting with muscle-invasive bladder cancer (MIBC) at diagnosis (1, 2). MIBC is associated with significantly reduced survival and radical cystectomy with pelvic lymph node dissection represents the only potentially curative treatment. International guidelines recommend neoadjuvant cisplatin-based chemotherapy in eligible patients, as neoadjuvant chemotherapy (NAC) yields an 18% reduction in mortality and an 8% improvement in 5-year survival (3, 4), with even greater benefit by also adding perioperative immunotherapy (5).

Accurate staging is paramount, especially the detection of nodal disease, which is usually the first way of metastatic spread and dramatically impacts prognosis, with 5-year overall survival of 45%−50% in N+ vs. 70%−75% in N0 patients (6, 7). However, conventional imaging approaches—including contrast-enhanced Computed Tomography (ceCT) and pelvic Magnetic Resonance Imaging (MRI)—suffer from low and variable sensitivity (9%−41%) for detecting nodal metastases confirmed by histopathology after cystectomy (8–11). Suboptimal staging bears the risk of underestimating disease extent and submitting patients with occult metastatic disease to major surgery without clinical benefit. Recent studies have highlighted the potential incremental value of 2-deoxy-2-[^18^F]fluoro-D-glucose ([^18^F]FDG) Positron Emission Tomography/Computed Tomography (PET/CT) in detecting nodal and distant metastases in MIBC, with sensitivities between 33 and 47% and an ability to identify CT-occult nodal metastases in approximately 22% of patients (8–10, 12). Consequently, recent EAU guidelines mention [^18^F]FDG PET/CT as a potentially useful tool for staging MIBC (3).

Assessment of response to neoadjuvant treatment remains an unmet need. Pathologic complete response (pCR) defined as the complete absence of residual tumor at cystectomy (ypT0, ypN0) is strongly associated with improved survival; however, to date no imaging modality has been validated to reliably evaluate treatment response (13–15). Limited evidence suggests that interim [^18^F]FDG PET/CT may predict pCR and identify chemosensitive tumors. Soubra and colleagues (16) reported a sensitivity and specificity of 75% and 89.7%, respectively, in predicting pCR at histological examination after radical cystectomy combined with pelvic lymphadenectomy. Furthermore, [^18^F]FDG PET/CT was able to identify chemosensitive tumors (defined as down-staging from MIBC to non-MIBC) after neoadjuvant chemotherapy, with a sensitivity of 83% and specificity of 94%. In the meta-analysis by Ko et al. (6), the reported sensitivity and specificity values of [^18^F]FDG PET/CT were 68% and 77%, respectively. Yet, broader validation is lacking.

A major technical limitation of [^18^F]FDG PET/CT in bladder cancer lies in urinary tracer accumulation, hindering visualization of the bladder wall on standard 60-min images (13). Early dynamic pelvic acquisitions have shown promising accuracy for T-stage evaluation and are correlated with histological grade and muscle invasion (17–19). Optimizing and validating this acquisition protocol could enhance the diagnostic utility of [^18^F]FDG PET/CT in MIBC.

Radiomics have emerged as powerful tool capable of extracting quantitative information from diagnostic images, hidden to the human eye (20). The implementation of these additional data into artificial intelligence (AI) models is being investigated in several clinical settings (21, 22). These tools have the potential to improve prediction of staging, response to therapy, and clinical outcomes, while sparing additional radiation exposure. Indeed, while scientific research has been flourishing in the field of radiomics and AI, the translation of these tools to clinical practice is still lacking due to issues related to reproducibility (i.e., retrospective design) and standardization of imaging protocols (i.e., heterogeneous tomography and acquisition protocols) (23).

The MIBC-PET study was therefore designed to prospectively compare [^18^F]FDG PET/CT with ceCT for baseline staging and response assessment to neoadjuvant treatment, validate early dynamic PET imaging, and explore radiomic analysis and AI-based predictive modeling.

## 2 MIBC-PET design

MIBC-PET is a prospective, monocentric, no-profit study with paired imaging comparison, conducted at the Nuclear Medicine Unit of the Azienda Ospedaliero-Universitaria di Ferrara (Ferrara, Italy).

The study aims to define the diagnostic potential of [^18^F]FDG PET/CT in baseline staging and in the assessment of response to neoadjuvant treatment in patients with newly diagnosed MIBC. By directly comparing [^18^F]FDG PET/CT with gold standard imaging—thoraco-abdomino-pelvic ceCT—the study seeks to determine whether [^18^F]FDG PET/CT offers superior diagnostic accuracy in defining disease extent at presentation. In addition, the study will evaluate the ability of interim [^18^F]FDG PET/CT (performed after two cycles of neoadjuvant treatment) to predict pathological response to neoadjuvant treatment, thereby clarifying its role in guiding therapeutic decision-making. The results are expected to contribute to a clearer definition of the role of this imaging modality within the diagnostic algorithm and therapeutic management of these patients.

Beyond these primary aims, the study also intends to validate and standardize an early dynamic pelvic acquisition protocol for [^18^F]FDG PET/CT, specifically tailored to improve local staging of bladder cancer. This approach will allow the characterization of tracer kinetics at the bladder wall, the biodistribution of radioactive urine, and the identification of the optimal time window for lesion evaluation without interference from urinary activity. Semi-quantitative parameters derived from early dynamic imaging will be analyzed to assess their diagnostic contribution.

Furthermore, the study will develop predictive models based on clinical data and radiomic features extracted from both [^18^F]FDG PET/CT and ceCT imaging. These models, trained through machine learning (ML) techniques, are expected to provide clinicians with advanced tools to anticipate study endpoints for individual patients, thereby supporting a personalized medicine approach.

### 2.1 Study population

Eligible patients are adults aged 18 years or older with a histologically confirmed diagnosis of high-grade MIBC. Only patients without prior oncologic treatments will be considered and candidates must be eligible for platinum-based neoadjuvant treatment on clinical evaluation—including an ECOG performance status of 0–1, a creatinine clearance of at least 60 ml/min, and the absence of comorbidities that would contraindicate chemotherapy such as grade ≥2 neuropathy, grade ≥2 hearing impairment, or significant cardiopathy. All participants must provide written informed consent before enrollment.

Patients will be excluded if they present with non–muscle-invasive bladder cancer, if they suffer from claustrophobia or are otherwise unable to undergo the required imaging procedures (i.e., history of iodinated contrast medium allergy precluding ceCT, or diabetes insufficiently controlled by medication). Inclusion and exclusion criteria are listed in Table 1.

**Table 1:** Inclusion and exclusion criteria for the MIBC-PET study.

**Inclusion criteria**	**Exclusion criteria**
Adults aged 18 years or older	Previously known oncological disease
Histologically confirmed diagnosis of high-grade MIBC	Non–muscle-invasive, or low-grade bladder cancer
Eligible for platinum-based neoadjuvant treatment on clinical evaluation: •ECOG performance status of 0–1, •Creatinine clearance of at least 60 ml/min, •Absence of comorbidities that would contraindicate chemotherapy (such as grade ≥2 neuropathy, grade ≥2 hearing impairment, or significant cardiopathy).	Impossibility to perform imaging procedures, including •Claustrophobia. •History of iodinated contrast medium allergy precluding ceCT. •Diabetes uncontrolled by medications.
Sign of informed consent	–

The planned enrollment period is 30 months. Given that the first patient was screened on April 14, 2024, recruitment is expected to close at the end of 2026.

### 2.2 Study procedures

All imaging procedures are performed using a Biograph mCT Flow PET/CT system (Siemens, Healthcare GmbH, Erlangen, Germany). For the early dynamic pelvic PET acquisition, patients receive an intravenous injection of [^18^F]FDG at a dose of 3 MBq/kg, followed by immediate dynamic acquisition in list-mode for a total duration of 10 min. Partial frames are reconstructed at pre-defined intervals (1, 2, 3, 4, 5, 6, 8, 10 min), and the optimal frame reconstruction is selected as the last acquired before the appearance of radioactive urine in the bladder—which is arbitrarily defined as a SUVmean < 0.3 within a 1 cm volume of interest placed in the bladder lumen.

In addition to the dynamic acquisition, a standard whole-body PET/CT scan is performed at 60 ± 10 min after tracer injection, covering the region from the skull base to the mid-thigh. Acquisition speeds are set at 1.1 mm/s for the pelvis, 1.3 mm/s for the abdomen and 1.5 mm/s for the thoracic and head and neck regions. A corresponding low-dose CT scan is acquired for attenuation correction (using a tube voltage of 120 kV with CARE Dose 4D modulation, a slice thickness of 5 mm, and a medium smooth B30F kernel filter, with images obtained in a caudo-cranial direction).

For diagnostic ceCT, a standardized CT urography protocol is employed. The examination includes a corticomedullary phase triggered at 150 Hounsfield Units, followed by a nephrographic phase acquired at approximately 80–100 s, and an excretory phase performed 7–10 min after contrast administration. The planned scanning parameters are as follows: cranio-caudal scan direction, 100–120 kVp, mAs modulation, 2 mm slice thickness. The contrast agent used is an iodinated non-ionic formulation, such as Iohexol at a concentration of 350 mg/ml. Administration of a contrast medium dose not exceeding 0.63 gI/Kg is planned.

**Figure 1:**
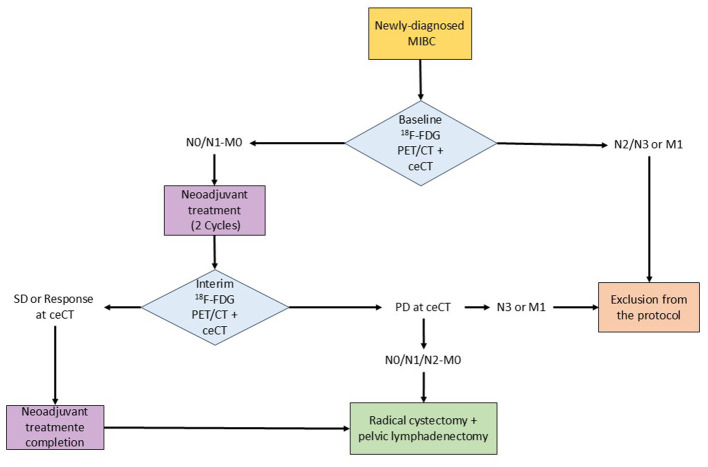
Flowchart of the study.

All patients enrolled in the study will perform the study imaging procedure at baseline and following two cycles of neoadjuvant treatment with cisplatin and gemcitabine, possibly in combination with perioperative durvalumab (interim) (5). Patients with evidence of *N* ≥ 2 or M1 disease at baseline scan will be excluded from the study. Conversely, N0/N1, M0 patients will be treated with neoadjuvant chemotherapy (with or without durvalumab) and scanned again after the completion of the 2nd cycle. In case of stable disease or response (partial or complete) at interim ceCT, patients will complete scheduled neoadjuvant treatment as per clinical protocol in use. Conversely, patients with evidence of local progression at ceCT will be directly addressed to surgery. Patients with extra pelvic lymph-nodes involvement or metastatic disease at interim imaging will exit the study protocol. The reference standard for diagnostic accuracy is provided by histopathological examination of specimens obtained from radical cystectomy and pelvic lymphadenectomy. pCR will be considered in case of complete absence of residual tumor at radical cystectomy (ypT0, ypN0). Tumors with evidence of down-staging from MIBC to non-MIBC after neoadjuvant treatment will be defined as chemosensitive. In cases of metastatic disease ruling out surgery, ceCT findings will serve as the reference standard, since ceCT is currently considered the gold standard imaging procedure for staging MIBC patients by EAU guidelines (3). Being an observational study, the choice of neoadjuvant treatment regimen follows routine clinical practice and may introduce heterogeneity, particularly if new medications (e.g., durvalumab) are incorporated during the study period. The study flowchart is reported in Figure 1.

### 2.3 Image analysis

All [^18^F]FDG PET/CT examinations will be independently reviewed by two nuclear medicine physicians, each with at least 5 years of experience in interpreting oncologic [^18^F]FDG PET/CT studies. In the event of discordant findings, a consensus will be reached by discussion. For every [^18^F]FDG PET/CT scan, a systematic and comprehensive evaluation will be conducted to identify all areas of pathological radiotracer uptake, defined as uptake exceeding background activity and occurring outside the expected physiological distribution of [^18^F]FDG.

Image interpretation will include assessment of the T–stage, focusing on the primary bladder tumor or, when the lesion has been resected at cystoscopy, on the resection site to exclude local recurrence. This evaluation will rely primarily on the early dynamic pelvic acquisition. N–stage assessment will include identification and characterization of pathological pelvic lymph nodes, documenting their number, anatomical location, and a reference lesion defined as the most representative node in terms of uptake intensity. M–stage evaluation will include detection of distant metastases, again recording number, location, and a reference lesion. In interim studies, treatment response will be assessed according to PERCIST 1.0 criteria (24).

For each pathological finding across all anatomical regions, semiquantitative PET parameters will be collected, including maximum, mean and peak Standardized Uptake Values (SUVmax, SUVmean and SUVpeak, respectively), metabolic tumor volume (MTV), and total lesion glycolysis (TLG), both on early and delayed imaging. MTV will be calculated using a threshold of 41% of the SUVmax within the volume of interest (VOI), and TLG will be obtained by multiplying MTV by the SUVmean of the same VOI.

CT images will be reviewed using the local Radiological Information System (RIS). Contrast-enhanced and non-contrast CT images will be independently evaluated by two radiologists with at least 5 years of experience in uro-CT interpretation, applying RECIST 1.1 criteria (25). Any discrepancies will be resolved through consensus discussion. The non-contrast CT phase acquired prior to PET will be used to detect urinary tract calculi and to assist in characterizing contrast-enhanced findings, allowing differentiation between non-enhancing and enhancing lesions (no enhancement if the increase in attenuation from non-contrast to post-contrast images is < 10 HU; indeterminate if 10–20 HU; significant if >20 HU).

Imaging studies will be interpreted by both patient-based analysis and lesion-based analysis.

### 2.4 Sample size calculation and statistical analysis

For the sample–size calculation, we considered the expected sensitivities for the identification of lymph–node metastases using ceCT and [^18^F]FDG PET/CT. Given the wide variability reported in the literature, we adopted conservative estimates derived from the lower end of published ranges and from the mean values reported in key studies evaluating newly diagnosed muscle–invasive bladder cancer. Specifically, we assumed sensitivities of 0.20 for ceCT and 0.37 for [^18^F]FDG PET/CT. Using these values, and assuming a two–sided type I error of 5% and a statistical power of 80%, we calculated that a total sample size of 50 patients would be required (8–10). The sample size calculated for primary endpoint 1 is sufficient to achieve primary endpoint 2 with a power greater than 80% (14). Therefore, 60 consecutive patients undergoing [^18^F]FDG PET/CT for the purposes of the present study will be included, accounting for an expected dropout rate of 10%−15% during recruitment (e.g., non-usable imaging, motion-degraded studies, examinations not performed due to comorbidities, etc.).

Based on detecting a 20% clinically relevant increase in diagnostic accuracy with SD 35%, 49 patients are required for 80% power at α = 0.05. To account for 10%−15% attrition (motion artifacts, unusable scans, comorbidities), 60 patients will be enrolled. This sample also provides >80% power for the second primary endpoint.

Diagnostic accuracy will be assessed using sensitivity, specificity, positive predictive value (PPV), negative predictive value (NPV), and receiving operator characteristics (ROC) curves. Comparative analyses between PET/CT and ceCT will use McNemar's test and paired statistics.

Radiomic analysis will be performed in compliance with IBSI standardization and consistently with previous published research (21). Radiomic features will be extracted using the PyRadiomics library implemented within 3D Slicer. All images will undergo standardized preprocessing, including voxel resampling, intensity normalization, and discretization, to ensure reproducibility and harmonization across the dataset.

Volumes of interest (VOIs) will be manually segmented on both scans on the primary bladder lesion and on the most representative lymph node, defined as the node showing the highest uptake intensity on [^18^F]FDG PET. Segmentation will be performed on ceCT acquired during the nephrographic phase, and corresponding VOIs will also be delineated on PET images from the multimodal dataset. A minimum VOI volume threshold of 2 cm^3^ will be applied to ensure stability of second– and higher–order radiomic features and to minimize the impact of image noise, voxel discretization, and partial–volume effects. Irregularly shaped tumors will not be excluded; instead, the minimum–volume criterion will ensure adequate voxel representation for reliable texture analysis. All VOIs will be visually inspected to confirm segmentation accuracy.

Radiomic feature extraction will include first–order statistics, shape descriptors, and texture–based features. Predictive modeling will integrate both clinical variables (e.g. smoking habit, presence of hematuria, date of diagnosis, tumor location within the bladder) and quantitative imaging–derived metabolic parameters. Feature selection will be performed using methods such as LASSO, U–type tests, PCA, and ANOVA. Multiple machine–learning classifiers—including Random Forest (RF), Support Vector Machine (SVM), and K–Nearest Neighbors (KNN)—will be trained and compared. Model performance will be evaluated using cross–validation, and the DeLong test will be applied to compare ROC curves and identify the best–performing algorithm.

Given the limited sample size, the radiomic analysis will be considered exploratory. A simplified approach (e.g., first–order features of the primary lesion on CT) will be evaluated as a sensitivity analysis, and findings will inform the design of a future multicenter study with larger cohorts and external validation.

### 2.5 Ethical considerations

The study has been approved by the local Ethics Committee (CE-AVEC 109-2024-Oss-AOUFe). The study complies with the Declaration of Helsinki, Good Clinical Practice guidelines, and all applicable national and European regulations. Written informed consent is obtained from all participants. Patient data will be anonymized using unique patient identifiers and entered into an electronic case report form (CRF). Only investigators have database access.

## 3 Discussion

MIBC is an aggressive cancer, still severely hampered today by reduced life expectancy in patients with metastatic disease at onset. Literature suggests that [^18^F]FDG PET/CT provides superior diagnostic accuracy compared with ceCT for baseline staging of MIBC, likely owing to its enhanced ability to stratify patients according to their true disease burden (8, 9, 12). The MIBC–PET study will provide the first prospective, systematic, head–to–head comparison between the two imaging modalities, with the potential to confirm or refute the superiority suggested by previous evidence and to inform evidence-based clinical decision-making. These findings may have substantial clinical implications. In particular, the availability of a more accurate staging method could enable more stringent patient selection, preventing patients with occult metastases—undetected on ceCT—from undergoing unnecessary radical surgery.

For neoadjuvant treatment response assessment, existing evidence is limited and inconsistent (14, 16). Interim [^18^F]FDG PET/CT may prove more accurate than ceCT in identifying metastatic spread of disease. Moreover, early dynamic [^18^F]FDG PET/CT acquisition could overcome longstanding technical limitations related to urinary tracer excretion, improving primary tumor chemo-sensibility assessment. The early PET scan could enable early identification of non-responders, allowing timely adjustment of treatment strategy. Indeed, recent literature indicates that MRI with the use of Vesical Imaging-Reporting And Data System (VI-RADs) criteria is the best imaging modality for local staging of MIBC, with potential application for response assessment after neoadjuvant treatment (weak recommendation according to EAU guidelines) (3, 15, 26). However, we selected thoraco–abdominal contrast–enhanced CT as the gold–standard imaging modality for comparison with [^18^F]FDG PET/CT, as it is recommended by the EAU guidelines for patients with histologically confirmed MIBC and provides the same whole–body field of view as PET imaging, allowing detection of distant metastases that would lie outside the field of view of MRI (3).

Radiomic and AI analyses may reveal imaging biomarkers that are imperceptible to the human eye, providing predictive tools that could enhance personalized management without adding further radiation burden to the patient (20). Research activity in this field has expanded considerably in recent years. However, the clinical implementation of radiomic and AI models remains limited by the need for greater methodological reproducibility and standardization (23). A major limitation of the current literature is that most radiomic studies rely on retrospective designs. Although monocentric and based on a medium–sized cohort, our study incorporates multimodal imaging ([^18^F]FDG PET/CT and ceCT) within a prospective framework. As such, it may represent an important foundation for the development of clinically and radiomics–based AI models, which can be externally validated in future research initiatives.

Finally, PET imaging has evolved rapidly in recent years, with several experimental radiotracers now being investigated in bladder cancer. While our study may help consolidate the scientific evidence supporting the role of [^18^F]FDG PET/CT, it may also serve as a foundation for future research exploring emerging PET agents, such as radiolabelled fibroblast activation protein inhibitors (FAPi), ^64^CuCl_2_ and nectin-4-targeting radiotracers, which hold promise for improving tumor characterization and expanding the molecular imaging landscape in MIBC (27–30).

In conclusion, the MIBC–PET study is designed to generate high–quality prospective data on the role of [^18^F]FDG PET/CT in baseline staging and response assessment during neoadjuvant treatment in newly diagnosed MIBC patients. Our aim is to provide a robust methodology aimed at demonstrating the superior diagnostic accuracy of [^18^F]FDG PET/CT compared with ceCT for the assessment of lymph node and distant metastases, as well as the non–inferiority of early [^18^F]FDG PET/CT pelvic imaging for evaluating local tumor extension and chemosensitivity in primary bladder cancer. In addition, contemporary radiomic analyses will be explored to further enhance prognostic stratification and support increasingly personalized clinical decision–making.
